# Efficacy and safety of Vibegron for the treatment of residual overactive bladder symptoms after laser vaporization of the prostate: A single‐center prospective randomized controlled trial (VAPOR TRIAL)

**DOI:** 10.1111/luts.12529

**Published:** 2024-07-02

**Authors:** Narihito Seki, Hiroyuki Masaoka, Yoohyun Song, Takashi Dejima, Yoshiaki Sato, Shotaro Maeda

**Affiliations:** ^1^ Department of Urology Kyushu Central Hospital of the Mutual Aid Association of Public School Teachers Fukuoka Japan; ^2^ Medical Affairs Kyorin Pharmaceutical Co, Ltd Tokyo Japan

**Keywords:** benign prostatic hyperplasia, overactive urinary bladder, transurethral resection of prostate

## Abstract

**Objectives:**

This study aimed to evaluate the efficacy and safety of Vibegron for the treatment of residual overactive bladder (OAB) symptoms after laser vaporization of the prostate (photo‐selective vaporization of the prostate, contact laser vaporization of the prostate, and thulium laser vaporization).

**Methods:**

This randomized, open‐label, parallel‐group, single‐center superiority trial with a 12‐week observation (jRCTs071190040) enrolled male patients with OAB aged 40 years or older who had undergone laser vaporization of the prostate for not less than 12 weeks and not more than 1 year earlier. Patients were allocated to receive Vibegron 50 mg once daily or follow‐up without treatment for 12 weeks.

**Results:**

Forty‐seven patients were enrolled between January 2020 and March 2023. The median age (interquartile range) was 75.5 (72.5–78.5) years for the Vibegron group and 76.5 (71.0–81.0) years for the control group. The intergroup difference in the mean change (95% confidence interval) in the 24‐hour urinary frequency at 12 weeks after randomization was −3.66 (−4.99, −2.33), with a significant decrease for the Vibegron group. The Overactive Bladder Symptom Score, International Prostate Symptom Score, IPSS storage score, and Overactive Bladder Questionnaire score significantly improved for the Vibegron group. Voided volume per micturition also increased for the Vibegron group.

**Conclusions:**

The administration of 50 mg of Vibegron once daily for 12 weeks showed significant improvement compared with follow‐up without treatment in bladder storage (OAB) symptoms after laser vaporization of the prostate for symptomatic benign prostatic hyperplasia.

## 1 INTRODUCTION

Benign prostatic hyperplasia (BPH) is a progressive disease among men who are middle‐aged and older. The prostates surrounding the urethra become enlarged, leading to various forms of urinary dysfunction and reduced quality of life (QOL). The prevalence of BPH in Japan depends on the criteria for diagnosis and symptoms, but it has been reported as 2% for patients in their 40s and 50s, 6% for those in their 60s, and 12% for those in their 70s.^1^, ^2^ The exact mechanisms underlying enlargement of the prostate are not well understood, but the obvious risk factors include aging, genetics, lifestyle, obesity, hypertension, hyperglycemia, and dyslipidemia.^3^, ^4^, ^5^

The major treatments for BPH are pharmacotherapy, surgery, and conservative treatment, and surgery is becoming a more common option in Japan.^6^ Minimally invasive techniques for BPH surgery have been advancing. Vaporization using lithium borate lasers (PVP: photo‐selective vaporization of the prostate), diode lasers (CVP: contact laser vaporization of the prostate), and thulium lasers (ThuVAP: thulium laser vaporization of the prostate) are commonly used, and they are covered by health insurance in Japan. These laser procedures have been reported to significantly improve subjective and objective symptoms associated with urinary dysfunction.^7^, ^8^, ^9^, ^10^, ^11^, ^12^

Despite the advances in surgical techniques, overactive bladder (OAB) symptoms may persist after BPH surgery, and QOL may not fully improve. However, there are no specific treatment recommendations for these patients in Japanese, European, or American guidelines, and further investigations are warranted.^13^, ^14^, ^15^, ^16^, ^17^

Several anticholinergics and β3 agonists have been introduced for the treatment of OAB symptoms. Vibegron is a novel, potent, and selective β3 agonist, and its efficacy for treating OAB has been determined in several clinical trials. It was first approved for clinical use in Japan (50 mg) in 2018^18^ and the FDA approved Vibegron (75 mg) in 2020.^19^ While Vibegron may be effective for treating residual OAB symptoms after BPH surgery, no studies have proven its effectiveness. In addition, previous studies of other β3 agonists and anticholinergics used only subjective measures such as the Overactive Bladder Symptom Score (OABSS) or International Prostate Symptom Score (IPSS).^20^, ^21^, ^22^

This study aimed to evaluate the efficacy and safety of Vibegron for treating residual OAB symptoms after laser vaporization of the prostate.

## 2 METHODS

### 2.1 Study design and patients

This was a single‐center, open‐label, randomized, parallel‐group comparative study. Patients included in the study were those who visited Kyushu Central Hospital between January 8, 2020, and March 31, 2023. Since postoperative lower urinary tract symptoms become stable approximately 3–12 months after vaporization,^12^ we enrolled patients who had undergone laser vaporization of the prostate (PVP, CVP, and ThuVAP) for at least 12 weeks but less than 1 year at the time of consent for participation in the study. The key inclusion criteria for this study were as follows: (1) men aged ≥40 years old with OAB (selection criteria: total OABSS^23^ ≥3 points and OABSS question 3 score ≥2 points); (2) OABSS question 1 score ≥1; (3) QOL index for IPSS (IPSS‐QOL) ≥2 points; (4) and mean 24‐h frequency of ≥8 times per day in the bladder diary at Week 0 were included. All inclusion and exclusion criteria are listed in Table S1.

Eligible patients were randomly assigned to the 50 mg of Vibegron once daily (approved dose in Japan) group (Vibegron group) or follow‐up without treatment group (follow‐up group) at baseline (0w). For randomization, a blinded central registry system was used, and a minimization method was applied with age (<65 or ≥65 years) and baseline 24‐hour frequency (<11 or ≥11) as adjustment factors. They visited the study center at 4, 8, and 12 weeks.

### 2.2 Outcomes

Patients completed a bladder diary 3 days before each visit at 0, 8, and 12 weeks for the assessment of the frequency of voiding. The OABSS, IPSS, IPSS‐QOL, and OAB Questionnaire (OAB‐q) score were assessed at 0, 4, 8, and 12 weeks, while the post‐void residual volume (PVR) and maximum flow rate (*Q*_max_) were assessed at 0 and 12 weeks.

The primary outcome of the efficacy assessment was the change in the mean 24‐h frequency from Weeks 0 to 12 for the Vibegron and follow‐up groups. The secondary outcomes were the inter‐group difference between the changes in the following from Weeks 0 to 12: urgency episodes, urgency incontinence episodes, night‐time frequency, and voided volume per micturition in the bladder diary, *Q*_max_, PVR, OABSS, IPSS, IPSS‐QOL, and OAB‐q. For the safety assessment, all adverse events that occurred during the study were included.

### 2.3 Statistical analyses

Based on the results of the Phase III study of Vibegron^18^ and an assumption of a mean difference of 1.5 between the 24‐h urinary frequencies of the Vibegron and placebo groups with a standard deviation (SD) of 2, the sample size was 28 patients for each group required for a two‐tailed test with a significance level of 5% and a power of 80%. To account for the number of dropouts during the study, the number of patients in each group was set at 30 for a total of 60 patients.

The efficacy assessment and analyses were performed on the Full Analysis Set (FAS); this covered all patients who were randomized, excluding those who withdrew their consent from the study. The safety assessment was performed on the Safety Set (SS), which covered all patients who were enrolled, excluding those who withdrew their consent from the study. The protocol‐compliant patients were also assessed for efficacy (PPS: Per Protocol Set).

For the primary outcome, which was the superiority of the Vibegron group over the follow‐up group, a constrained Longitudinal Data Analysis (cLDA) model including age at consent, visit, and the interaction between visit and treatment group was used to estimate the least squares means, 95% confidence intervals (95% CI), and *p‐*values for the change in the mean 24‐h frequency from Weeks 0 to week 12. The cLDA model was also applied to the secondary outcomes analyses for each variable. A multivariable logistic regression model was applied with age at consent and baseline scores as adjustment factors to compare the proportion of patients achieving minimally clinically important change (MCIC)^24^ on the OABSS and minimally important difference (MID)^25^ on the OAB‐q between the groups. Furthermore, multivariable analysis was performed to explore the background factors that were associated with the change in the mean 24‐h frequency at 12 weeks. In this analysis, univariable analysis was initially performed using the patient background factors (only total score for OABSS, IPSS, and OAB‐q) and allocation adjustment factors such as age at consent and mean 24‐h frequency as independent variables, followed by multivariable analysis where factors with *p*‐values less than .2 were included in the model as independent variables.

The baseline patient background characteristics of each group in the FAS are summarized as mean (SD) or median (interquartile range [IQR]) for continuous variables and frequency and proportion (%) for categorical variables. The differences in distribution between the groups are also evaluated by the Wilcoxon rank‐sum test or chi‐squared test. All statistical tests were two‐tailed with a significance level of 5%, and all analyses were performed using SAS software version 9.4 (SAS Institute, Cary, NC, USA).

### 2.4 Ethics statement

This study was conducted following the ethical principles of the Declaration of Helsinki and in compliance with the Clinical Trials Act.^26^ It was approved by Kyushu University Certified Institutional Review Board for Clinical Trials (jRCTs071190040). Written informed consent was obtained from all patients before the study enrollment.

## 3 RESULTS

### 3.1 Patient disposition and baseline characteristics

**FIGURE 1:**
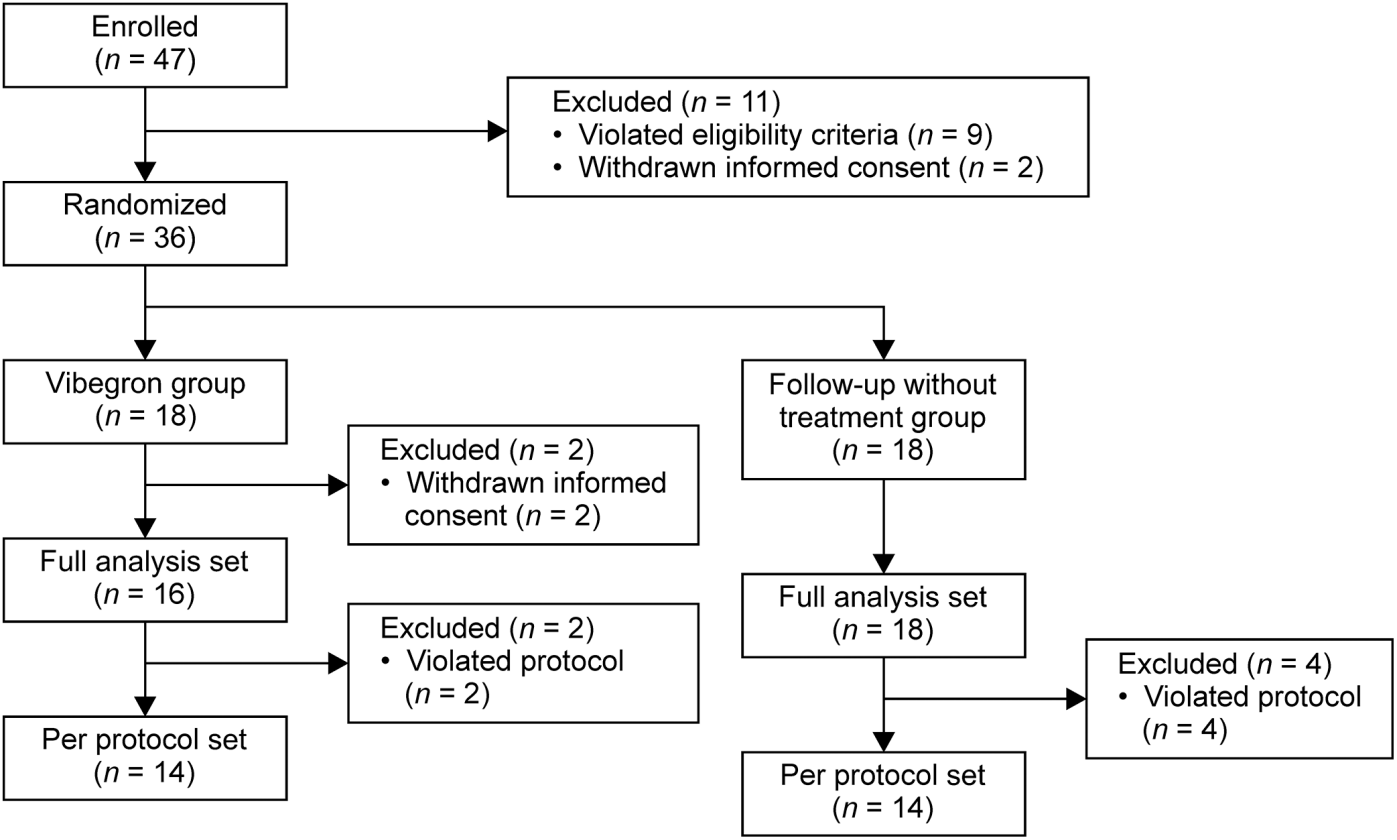
Patient disposition.

This study was conducted between January 8, 2020, and March 31, 2023, at Kyushu Central Hospital in Japan. Informed consent was obtained from 47 patients, and 36 patients were randomly assigned to the study group according to eligibility criteria (Figure 1). Of the 36 randomized patients, 34 were included in the FAS and SS, excluding 2 patients who withdrew their consent. The baseline patient characteristics for the FAS population were similar for the Vibegron and follow‐up groups (Table 1). The mean ages (SD) of the patients in the Vibegron and follow‐up groups were 76.2 (6.7) and 75.3 (7.6) years, respectively, and the vaporization techniques applied were PVP for 7 (43.8%) and 8 (44.4%) patients and CVP for 9 (56.3%) and 10 (55.6%) patients, respectively. There were no differences in the background characteristics, except the night‐time frequency of voiding.

**TABLE 1:** Patient characteristics at baseline.

	Vibegron group	Follow‐up group	*p*‐Value*
Variables	*n* = 16	*n* = 18	
Age, years			
Mean (SD)	76.2 (6.7)	75.3 (7.6)	.986
BMI, kg/m^2^			
Mean (SD)	23.7 (3.9)	23.7 (3.4)	.904
Type of vaporization, *n* (%)			.968
PVP	7 (43.8%)	8 (44.4%)	
CVP	9 (56.3%)	10 (55.6%)	
Thu VAP	0 (0.0%)	0 (0.0%)	
Duration from vaporization, days			
Mean (SD)	179.0 (81.2)	184.7 (98.9)	.856
Duration of OAB symptoms, months			
Mean (SD)	5.0 (6.8)	8.5 (22.2)	.786
Onset of OAB symptoms, *n* (%)			
After vaporization	7 (43.8%)	11 (61.1%)	.504
Comorbidity, *n* (%)			
Diabetes	3 (18.8%)	1 (5.6%)	.233
Hypertension	7 (43.8%)	9 (50.0%)	.716
Constipation	2 (12.5%)	1 (5.6%)	.476
Cardiovascular disease	0 (0.0%)	1 (5.6%)	.339
Urinary symptom‐related variables, mean (SD)			
Bladder diary			
24‐h frequency	12.6 (3.2)	11.4 (3.0)	.112
24‐h urgency episodes	4.4 (4.1)	2.0 (2.2)	.156
24‐h urgency incontinence episode	0.3 (0.6)	0.1 (0.3)	.285
Night‐time frequency	3.0 (1.4)	2.1 (0.9)	.025
Voided volume/micturition, mL	168.6 (53.7)	150.7 (43.5)	.593
OABSS total score	7.8 (2.0)	7.1 (1.9)	.302
IPSS total score	11.9 (4.1)	12.1 (6.3)	.691
Voiding score	3.2 (2.4)	4.3 (3.8)	.444
Storage score	7.8 (2.4)	6.8 (2.9)	.339
IPSS‐QOL score	4.0 (1.1)	3.8 (1.3)	.654
OAB‐q total score	80.4 (25.9)	74.9 (23.2)	.458
Symptom bother	25.3 (10.1)	20.4 (7.9)	.129
HRQoL	55.2 (17.0)	54.5 (17.0)	.907
PVR, mL	19.8 (21.2)	11.2 (18.8)	.109
*Q*_max_, mL/s	12.6 (6.9)	11.3 (9.1)	.333
Prostate volume, mL	36.8 (16.2)	31.1 (16.3)	.384

Abbreviations: BMI, body mass index; CVP, contact laser vaporization of the prostate; HRQoL, health‐related quality of life; IPSS, International Prostate Symptom Score, IPSS‐QOL, quality of life index in IPSS, OAB‐q, Overactive Bladder Questionnaire; OABSS, Overactive Bladder Symptom Score; PVP, photo‐selective vaporization of the prostate; PVR, post‐void residual volume; *Q*_max_, maximal flow rate; ThuVAP, thulium laser vaporization of the prostate.

* *p*‐values were derived from the Wilcoxon rank‐sum test for continuous variables and the chi‐square test for categorical variables.

### 3.2 Analyses of primary and secondary outcomes

**FIGURE 2:**
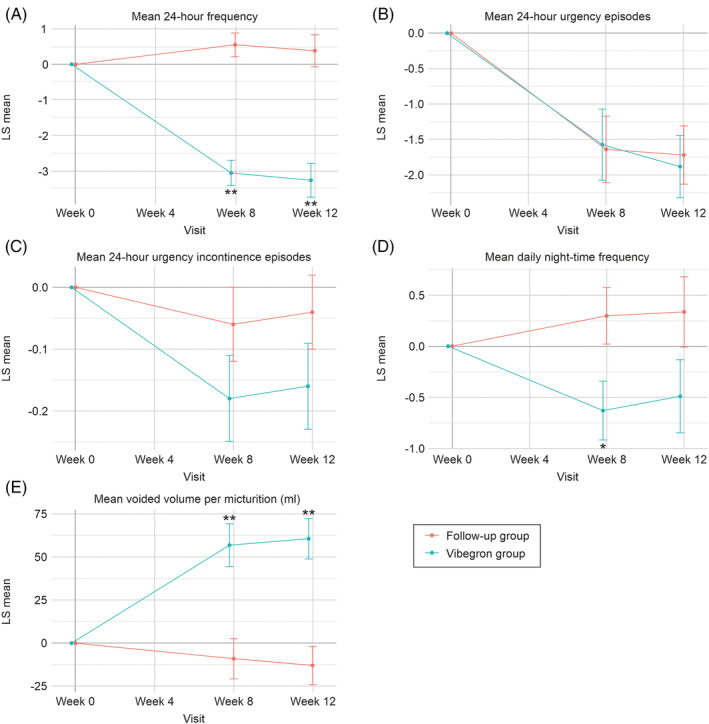
Least‐square (LS) means ± SE from baseline at each visit. (A) Mean 24‐h frequency. (B) Mean 24‐h urgency episodes. (C) Mean 24‐h urgency incontinence episodes. (D) Mean daily night‐time frequency. (E) Mean voided volume per micturition (mL). *p*‐values were derived from cLDA model. *p < .05 (vs. follow‐up group), **p < .001 (vs. follow‐up group).

The primary outcome, which was the change in the mean 24‐h frequency from week 0 to week 12 for the FAS was −3.28 (95% CI: −4.25, −2.32) for the Vibegron group and 0.38 (95% CI: −0.53, 1.29) for the follow‐up group. The difference between the groups was −3.66 (95% CI: −4.99, −2.33; *p* < .001 vs. follow‐up group), indicating a significant improvement for the Vibegron group, relative to that of the follow‐up group (Figure 2, Table 2). Similar results were observed for the PPS (Table S2). In addition, the change in the mean 24‐h frequency from Weeks 0 to 8 was −3.07 (95% CI: −3.80, −2.35) for the Vibegron group and 0.55 (95% CI: −0.14, 1.23) for the follow‐up group, with a significant difference of −3.62 (95% CI: −4.62, −2.61; *p* < .001 vs. follow‐up group) (Table 2).

**TABLE 2:** Comparisons of the primary and secondary outcomes of the groups (changes from Weeks 0 to 12).

	Groups						Difference between groups		*p*‐Value*
	Vibegron			Follow‐up					
Variables	*n*	LSM	SE	*n*	LSM	SE	LSM	95% CI	
Bladder diary									
24‐h frequency									
Week 8	16	−3.07	0.35	18	0.55	0.33			
Week 12	16	−3.28	0.47	18	0.38	0.45	−3.66	[−4.99, −2.33]	<.001
24‐hour urgency									
Week 8	16	−1.57	0.50	18	−1.64	0.47			
Week 12	16	−1.88	0.44	18	−1.72	0.41	−0.17	[−1.44, 1.11]	.792
24‐hour urgency incontinence episode									
Week 8	16	−0.18	0.07	18	−0.06	0.06			
Week 12	16	−0.16	0.07	18	−0.04	0.06	−0.12	[−0.30, 007]	.212
Night‐time frequency									
Week 8	16	−0.63	0.29	18	0.30	0.27			
Week 12	16	−0.49	0.36	18	0.34	0.34	−0.83	[−1.86, 0.21]	.114
Voided volume/micturition, mL									
Week 8	16	56.76	12.40	18	−8.97	11.68			
Week 12	16	60.55	11.72	18	−12.76	11.04	73.31	[40.12, 106.50]	<.001
OABSS total score									
Week 4	15	−2.47	0.63	18	−0.99	0.59			
Week 8	16	−3.08	0.58	18	−1.15	0.54			
Week 12	16	−3.39	0.46	18	−1.99	0.43	−1.40	[−2.70, −0.10]	.035
IPSS total score									
Week 4	15	−3.04	0.97	18	−1.61	0.89			
Week 8	16	−4.37	0.89	18	−.0.78	0.84			
Week 12	16	−4.50	0.85	18	−.2.00	0.80	−2.49	[−4.89, −0.10]	.042
IPSS voiding score									
Week 4	15	−0.32	0.57	18	−0.87	0.53			
Week 8	16	−0.47	0.46	18	−0.15	0.43			
Week 12	16	−0.97	0.43	18	−1.32	0.41	0.35	[−0.87, 1.56]	.565
IPSS storage score									
Week 4	15	−2.47	0.63	18	−0.90	0.59			
Week 8	16	−3.58	0.59	18	−0.73	0.55			
Week 12	16	−3.21	0.51	18	−1.06	0.48	−2.14	[−3.59, −0.69]	.005
IPSS‐QOL									
Week 4	15	−0.95	0.35	18	−0.67	0.32			
Week 8	16	−1.36	0.32	18	−0.56	0.30			
Week 12	16	−1.05	0.30	18	−0.34	0.29	−0.71	[−1.56, 0.15]	.102
OAB‐q total score									
Week 4	15	−20.21	3.89	18	−5.81	3.60			
Week 8	16	−26.38	3.98	18	−7.54	3.75			
Week 12	16	−29.50	4.30	18	−7.31	4.05	−22.19	[−34.27, −10.11]	<.001
OAB‐q symptom bother									
Week 4	15	−7.80	1.09	18	−2.31	1.00			
Week 8	16	−9.22	1.27	18	−2.86	1.20			
Week 12	16	−10.54	1.55	18	−2.48	1.46	−8.06	[−12.44, −3.68]	<.001
OAB‐q HRQoL									
Week 4	15	−12.50	3.21	18	−3.51	2.97			
Week 8	16	−17.15	3.01	18	−4.68	2.84			
Week 12	16	−18.96	3.07	18	−4.84	2.89	−14.11	[−22.73, −5.49]	.002

Abbreviations: CI, confidence interval; HRQoL, health‐related quality of life; IPSS, International Prostate Symptom Score, IPSS‐QOL, quality of life index in IPSS; LSM, least square means; OAB‐q, Overactive Bladder Questionnaire; OABSS, Overactive Bladder Symptom Score; SE, standard error.

* *p*‐values were derived from the cLDA model.

The secondary outcomes of the Vibegron and follow‐up groups differed significantly and were as follows: the mean voided volume per micturition increased by 73.31 (95% CI: 40.12, 106.50; *p* < .001). The OABSS total score, −1.40 (95% CI: −2.70, −0.10; *p* = .035); IPSS total score, −2.49 (95% CI: −4.89, −0.10; *p* = .042); IPSS storage score, −2.14 (95% CI: −3.59, −0.69; *p* = .005); and OAB‐q total score, −22.19 (95% CI: −34.27, −10.11; *p* < .001) (Table 2). Regarding the proportion of patients in each group who achieved the MCIC of 3 points decrease on the OABSS total score, 11 (68.8%) and 8 (44.4%) patients achieved this improvement in the Vibegron and follow‐up groups, respectively, with no significant between‐group difference (*p* = .256) (Table S3). Furthermore, regarding the proportion of patients achieving the MID (10‐point reduction) in OAB‐q, 9 (56.3%) and 2 (11.1%) patients in the Vibegron and follow‐up groups, respectively, achieved an MID in symptom bother, and 11 (68.8%) and 6 patients in the Vibegron and follow‐up groups, respectively, achieved a MID in health‐related quality of life (HRQoL), with significant between‐group differences (*p* = .031 and *p* = .042, respectively) (Table S3).

### 3.3 Background factors associated with the primary outcome

The background factors associated with the change in the 24‐h frequency from Weeks 0 to 12 were determined using secondary outcome analysis. Univariate analysis showed that the baseline variables with *p*‐values less than .2 were the treatment group (*p* < .001), mean daily voiding episode (*p* = .078), diabetes mellitus (*p* = .041), urinary urgency episode (*p* = .089), urgency urinary incontinence episode (*p* = .102), and night‐time frequency (*p* = .022). Multivariable analysis with these factors as independent variables showed that only the treatment group was significantly associated with the change in the 24‐h frequency from Weeks 0 to 12 (*p* < .001) (Table 3). For the safety assessment, three adverse events occurred in two patients in the follow‐up group (one case of constipation and two cases of other adverse events). No adverse events were recorded for the Vibegron group.

**TABLE 3:** Univariable and Multivariable analyses of the factors associated with the mean 24‐h frequency.

		Univariable analysis				Multivariable analysis			
Variables	Reference	*n*	Estimate	95% CI	*p*‐Value*	*n*	Estimate	95% CI	*p*‐Value*
Group—Vibegron group	Follow‐up group	16/18	−3.68	[−4.99, −2.38]	<.001	16/18	−3.39	[−4.89, −1.88]	<.001
Age, years		34	0.01	[−0.18, 0.14]	.933	34	0.01	[−0.10, 0.12]	.853
BMI, kg/m^2^		34	−0.15	[−0.41, 0.10]	.234				
Duration of OAB symptoms		34	−0.01	[−0.06, 0.05]	.923				
Comorbidity									
Diabetes	No	4/30	−2.82	[−5.52, −0.13]	.041	4/30	−1.62	[−4.23, 0.10]	.213
Hypertension	No	16/18	0.69	[−1.16, 2.53]	.453				
Constipation	No	3/31	−1.57	[−4.80, 1.65]	.328				
Cardiovascular disease	No	1/33	2.41	[−3.01, 7.84]	.372				
Vaporization–CVP	PVP	19/15	−0.06	[−1.93, 1.81]	.951				
OABSS		34	−0.17	[−0.66, 0.32]	.483				
IPSS		34	0.05	[−0.13, 0.22]	.597				
IPSS‐QOL		34	−0.36	[−1.13, 0.42]	.353				
OAB‐q		34	−0.01	[−0.04, 0.04]	.823				
Urinary diary									
24‐h frequency		34	−0.26	[−0.55, 0.03]	.078	34	−0.14	[−0.41, 0.14]	.314
24‐h urgency episodes		34	−0.23	[−0.50, 0.04]	.089	34	0.10	[−0.15, 0.35]	.432
24‐h urgency incontinence episode		34	−1.69	[−3.72, 0.35]	.102	34	−0.60	[−2.28, 1.09]	.472
Night‐time frequency		34	−0.83	[−1.54, −0.13]	.022	34	−0.04	[−0.81, 0.74]	.917
Voided volume/micturition, mL		34	−0.01	[−0.02, 0.02]	.900				
PVR, mL		34	−0.02	[−0.07, 0.02]	.300				
*Q*_max_, mL/s		34	−0.02	[−0.14, 0.10]	.737				
PV, mL		31	−0.03	[−0.09, 0.03]	.322				

Abbreviation: BMI, body mass index; CI, confidence interval; IPSS, international prostate symptom score; IPSS‐QOL, quality of life index in IPSS; LSM, least square means; OAB‐q, overactive bladder questionnaire; OABSS, overactive bladder symptom score; PV, prostate volume; PVR, post‐void residual; *Q*_max_, maximum urine flow rate; SE, standard error.

* *p*‐Values were derived from the cLDA model.

## 4 DISCUSSION

This is the first prospective study to show that 50 mg of Vibegron once daily significantly improves residual storage symptoms after laser vaporization of the prostate in a randomized, controlled study. Relative to the follow‐up group, the Vibegron group demonstrated significant improvement in the primary outcomes, including the 24‐h frequency, as well as the secondary outcomes, such as voided volume per micturition, OABSS total score, IPSS total score, and OAB‐q total score. In addition, the exploratory analysis demonstrated that the treatment group was the only factor associated with the primary outcome.

Complications associated with transurethral resection and PVP techniques for BPH have been reported; they included intraoperative hemorrhage^27^ and postoperative deterioration of QOL due to sexual dysfunction and bladder irritation.^28^, ^29^ In a previous study that aimed to compare the efficacy and safety of PVP, CVP, and Thu VAP, the residual OAB measured by the OABSS total score was reported to be 4.9 (range, 1–15), 5.7 (range, 2–13), and 4.6 (0–12), respectively, at 3 months after surgery, suggesting that several patients had residual OAB symptoms even after laser vaporization for BPH.^12^ The evidence for drug therapy for postoperative OAB symptoms is limited; only a few studies have evaluated the efficacies of mirabegron, tolterodine, and solifenacin for radical prostatectomy.^20^, ^21^, ^22^ In these studies, subjective measures such as OABSS or IPSS were used, and they indicated improvement in some symptoms. Postoperative storage symptoms lead to a significant reduction of patient QOL,^30^ and effective treatment options are in demand. In this study, the primary outcome, which was the mean 24‐h frequency for the Vibegron group, decreased by 3.28 times from the baseline (mean age of patients: 75.3–76.2 years [males only]). This finding is not comparable to the −2.08 change in 24‐h frequency obtained in the Phase III study of Vibegron^18^ (mean age, 58.0–59.4 years, approximately 90% female); however, the results in this study were similar or greater. The results may contribute to the clinically meaningful improvement of postoperative symptoms associated with BPH.

For the secondary outcomes of the Vibegron group, the OABSS total score, IPSS total score, and IPSS storage score decreased by 3.39, 4.50, and 3.21 points, respectively, and the mean voided volume per micturition increased by 60.55 mL. Those changes were significant improvements relative to the follow‐up group. Goto et al. reported that the OABSS is useful for assessing the treatment of OAB symptoms and demonstrated that the MCIC in OABSS was −3.^24^ In this study, the change in the OABSS score for the Vibegron group exceeded −3, indicating that the effect of Vibegron on residual OAB after laser vaporization of the prostate is clinically significant. However, no significant difference was found between the Vibegron and follow‐up groups in urinary urgency and urge urinary incontinence, which are both important factors in OAB symptoms. A randomized trial conducted by Li et al.^20^ evaluating the effect of Mirabegron on post‐prostatectomy OAB symptoms revealed that 24‐h frequency was consistently significantly lower in the Mirabegron group than in the control group from 4 to 12 weeks after prostatectomy. However, the group difference in urgency and urge urinary incontinence at 4 weeks was narrowed at 12 weeks, similar to the results of the present study. Okada et al.^12^ reported that OAB symptoms tended to improve from 3 to 12 months after vaporization; therefore, the scores in both groups may have also tended to improve in this study, which could explain why no significant difference was found between the groups. Additionally, the lack of significant difference in 24‐h urgency incontinence episodes may be due to the relatively low baseline values of 0.3 and 0.1 in the Vibegron and follow‐up groups, respectively. Finally, the crude values of urinary urgency showed a larger change in the Vibegron group than in the follow‐up group (Supplemental Table S4), although the mean baseline values were also larger in the Vibegron group, suggesting that the estimates (least squares mean) were adjusted accordingly in the multivariable analysis.

The total score for the OAB‐q, which evaluates the symptom bother and health‐related quality of life (HRQoL), for OAB symptoms,^24^ also significantly improved for the Vibegron group relative to the follow‐up group, with a change of −22.19 (95% CI: −34.27, −10.11; *p* < .001). This indicated a significant HRQoL improvement due to an administration of 50 mg of Vibegron once daily. Finally, multivariable analysis was performed to explore the background factors associated with the change in the mean 24‐h frequency at 12 weeks. The treatment group was the only factor associated with the primary outcome. In conclusion, these results suggest that treatment with 50 mg of Vibegron once daily in patients with residual OAB symptoms after laser vaporization of the prostate is useful. However, there were no inter‐group differences between the changes in the mean daily urgency episodes, urgency incontinence episodes, night‐time frequency, IPSS voiding score, and IPSS‐QOL score from baseline to 12 weeks. For those variables, there may have been no room for detecting statistically significant differences because the number of cases or the baseline scores were relatively low (e.g., urgency incontinence episodes; 0.3 for the Vibegron group and 0.1 for the follow‐up group) (Table 1).

Regarding safety assessment, this study reported three adverse events (two patients) in the follow‐up group and none in the Vibegron group. In the Phase III study,^18^ which evaluated the efficacy and safety of Vibegron over the same 12‐week period, the overall incidence of adverse events was similar in the Vibegron of 50 mg/day group (7.6%) and placebo group (5.1%). Despite the difference in the age and sex of the patients in the Phase III study, the safety results were similar.

This study has several limitations. First, we were unable to include the initially planned number of patients due to the changes in the social environment caused by COVID‐19. This resulted in the comparison of a few patients from a single center. However, in the opinion of the study's statistician, despite the small sample size, subgroup analyses by background factors for the primary outcome showed no factors that resulted in considerably smaller differences between groups or showed opposite results, suggesting that background factor bias due to insufficient number of cases may have had minimal effect on the results (Table S5). Second, this was an unblinded, open study, and it cannot be denied that the effect of the intervention in the Vibegron group may have been overestimated. However, the results obtained from this study indicate a large difference between the two groups with a relatively narrow 95% confidence interval, suggesting that the results of this study are meaningful for interpretation.

## 5 CONCLUSION

This study demonstrated that 50 mg of Vibegron once daily for 12 weeks showed significant improvement compared with follow‐up without treatment in bladder storage (OAB) symptoms after laser vaporization of the prostate for symptomatic BPH.

## AUTHOR CONTRIBUTIONS

NS, HM, YS, TD, and YS contributed to the conception and design of this study and are responsible for the work and results. All authors prepared and reviewed the manuscript and agreed to submit it for publication. NS, HM, YS, TD, and YS were responsible for access to data and statistical analysis.

## FUNDING INFORMATION

This work was supported and funded by Kyorin.

## CONFLICT OF INTEREST STATEMENT

NS received research funding from Kyorin. SM is a full‐time employee of Kyorin.

## Supporting information


**Table S1.** Eligibility criteria.

**Table S2.** Comparison of the primary and secondary outcomes of the subgroups of the PPS group (changes from Weeks 0 to 12).

**Table S3.** Comparison of proportion of patients achieving MCIC for OABSS and MID for OAB‐q using multivariable logistic regression model.

**Table S4.** Change from baseline for each endpoint (crude value).

**Table S5.** Change in mean 24‐h frequency from baseline to 12 weeks in a population subgrouped by patients' background.

## Data Availability

Research data are not shared.
