# Supplementary material for: Efficacy and safety of Vibegron for the treatment of residual overactive bladder symptoms after laser vaporization of the prostate: A single‐center prospective randomized controlled trial (VAPOR TRIAL)
Source: Low Urin Tract Symptoms. 2024 Jul 2;16(4):e12529. doi: 10.1111/luts.12529 (PMC11500685; doi:10.1111/luts.12529)
Supplement: Supplementary file 1 — Table S1. Eligibility criteria. [file LUTS-16-e12529-s004.docx]

Table S1 Eligibility criteria

| Inclusion criteria | Exclusion criteria |
| --- | --- |
| Patients who met all of the following criteria at the time of confirmation.   - Male, aged ≥ 40 years - Patients who had undergone laser prostate vaporization (PVP, CVP, or Thu VAP) at least 12 weeks and less than 1 year at the time of informed consent - Patients with IPSS-QOL ≥ 2 points - Patients with OABSS ≥ 3 points, Q1 ≥ 1 point, and Q3 ≥ 2 points - Patients who were able to keep a urinary diary - Patients who have given written consent - Patients with mean daily voiding episodes of 8 or more times per day at week 0 | Patients who met any of the following criteria at the time of confirmation of selection criteria were excluded from the study.   - PVR ≥ 100 mL - Patients with prolonged catheterization after laser prostate vaporization technique (PVP, CVP, or Thu VAP) or administered intermittent self-catheterization - Patients with comorbidities that present similar symptoms to those of OAB (diabetes insipidus, urinary tract infection, interstitial cystitis, and prostatitis) and are considered by the principal investigator or sub-investigator to negatively affect the evaluation of this study - Patients with only stress urinary incontinence - Patients with a history of injury, surgery, or neurodegenerative disease (multiple sclerosis) affecting the lower urinary tract and innervation. - Patients with bladder or prostate cancer who were undergoing or scheduled to undergo radiotherapy - Patients who had already received treatment for OAB - Patients who had received drugs affecting lower urinary tract symptoms (alpha 1 blockers, PDE5 inhibitors, anticholinergics, or beta 3 agonists) within the last 2 months - Patients who had received 5-alpha reductase inhibitors within the last 3 months - Patients who are considered to be unsuitable to participate in this study by the principal investigator or sub-investigator (s) |

Abbreviations: CVP, contact laser vaporization of the Prostate; IPSS, International Prostate Symptom Score, IPSS-QOL, quality of life index in IPSS; OAB, Overactive Bladder; OABSS, Overactive Bladder Symptom Score; PDE5, phosphodiesterase 5; PVP, photo-selective vaporization of the prostate; PVR, post-void residual volume; Thu VAP, thulium laser vaporization of the prostate
