# Supplementary material for: Efficacy and safety of Vibegron for the treatment of residual overactive bladder symptoms after laser vaporization of the prostate: A single‐center prospective randomized controlled trial (VAPOR TRIAL)
Source: Low Urin Tract Symptoms. 2024 Jul 2;16(4):e12529. doi: 10.1111/luts.12529 (PMC11500685; doi:10.1111/luts.12529)
Supplement: Supplementary file 2 — Table S2. Comparison of the primary and secondary outcomes of the subgroups of the PPS group (changes from Weeks 0 to 12). [file LUTS-16-e12529-s002.docx]

Table S2 Comparison of the primary and secondary outcomes of the subgroups of the PPS group (changes from week 0 to week 12)

|  | Groups | | | | | | | | | |  | Difference  between group | | | | *P*-value* | |  |  |
| --- | --- | --- | --- | --- | --- | --- | --- | --- | --- | --- | --- | --- | --- | --- | --- | --- | --- | --- | --- |
|  | Vibegron | | |  | Follow-up | | | | | |  |  |  |  |  |  |  |  |  |
| Variables | *n* | LSM | SE |  | *n* | | LSM | | SE | |  | LSM | | 95% CI | |  |  |  |  |
| Bladder diary |  |  |  |  |  | |  | |  | |  |  | |  | |  | |  |  |
| 24-hour frequency | | | | | | | | | | |  |  | |  | |  | |  |  |
| Week 8 | 14 | -3.07 | 0.41 |  | 14 | | 0.55 | | 0.41 | |  |  | |  | |  | |  |  |
| Week 12 | 14 | -3.43 | 0.56 |  | 14 | | 0.50 | | 0.56 | |  | -3.94 | | [-5.56, -2.31] | | <.001 | |  |  |
| 24-hour urgency | | | | | | | | | | |  |  | |  | |  | |  |  |
| Week 8 | 14 | -1.80 | 0.56 |  | 14 | | -1.77 | | 0.56 | |  |  | |  | |  | |  |  |
| Week 12 | 14 | -2.18 | 0.48 |  | 14 | | -1.79 | | 0.48 | |  | -0.39 | | [-1.82, 1.04] | | 0.582 | |  |  |
| 24-hour urgency incontinence episode | | | | | | | | | | |  |  | |  | |  | |  |  |
| Week 8 | 14 | -0.15 | 0.03 |  | 14 | | -0.16 | | 0.03 | |  |  | |  | |  | |  |  |
| Week 12 | 14 | -0.12 | 0.05 |  | 14 | | -0.11 | | 0.05 | |  | -0.01 | | [-0.17, 0.15] | | 0.910 | |  |  |
| Night-time frequency | | | | | | | | | | |  |  | |  | |  | |  |  |
| Week 8 | 14 | -0.57 | 0.32 |  | 14 | | 0.35 | | 0.32 | |  |  | |  | |  | |  |  |
| Week 12 | 14 | -0.66 | 0.39 |  | 14 | | 0.38 | | 0.39 | |  | -1.04 | | [-2.20, 0.11] | | 0.075 | |  |  |
| Voided volume/micturition, mL | | | | | | | | | | |  |  | |  | |  | |  |  |
| Week 8 | 14 | 60.31 | 14.10 |  | 14 | | -3.42 | | 14.10 | |  |  | |  | |  | |  |  |
| Week 12 | 14 | 62.55 | 13.48 |  | 14 | | -9.81 | | 13.48 | |  | 72.36 | | [32.72, 111.99] | | <.001 | |  |  |
| OABSS total score |  |  |  |  |  | |  | |  | |  |  | |  | |  | |  |  |
| Week 4 | 13 | -2.89 | 0.66 |  | 14 | | -0.61 | | 0.64 | |  |  | |  | |  | |  |  |
| Week 8 | 14 | -3.46 | 0.57 |  | 14 | | -0.90 | | 0.57 | |  |  | |  | |  | |  |  |
| Week 12 | 14 | -3.53 | 0.52 |  | 14 | | -1.90 | | 0.52 | |  | -1.63 | | [-3.17, -0.10] | | 0.038 | |  |  |
| IPSS total score |  |  |  |  |  | |  | |  | |  |  | |  | |  | |  |  |
| Week 4 | 13 | -3.04 | 1.08 |  | 14 | | -1.36 | | 1.06 | |  |  | |  | |  | |  |  |
| Week 8 | 14 | -4.92 | 0.94 |  | 14 | | -0.94 | | 0.94 | |  |  | |  | |  | |  |  |
| Week 12 | 14 | -4.63 | 0.95 |  | 14 | | -2.29 | | 0.95 | |  | -2.34 | | [-5.11, 0.43] | | 0.094 | |  |  |
| IPSS voiding score |  |  |  |  |  | |  | |  | |  |  | |  | |  | |  |  |
| Week 4 | 13 | 0.12 | 0.63 |  | 14 | | -0.50 | | 0.62 | |  |  | |  | |  | |  |  |
| Week 8 | 14 | -0.58 | 0.45 |  | 14 | | -0.29 | | 0.45 | |  |  | |  | |  | |  |  |
| Week 12 | 14 | -0.79 | 0.48 |  | 14 | | -1.36 | | 0.48 | |  | 0.57 | | [-0.84, 1.97] | | 0.412 | |  |  |
| IPSS storage score |  |  |  |  |  | |  | |  | |  |  | |  | |  | |  |  |
| Week 4 | 13 | -2.92 | 0.64 |  | 14 | | -0.84 | | 0.63 | |  |  | |  | |  | |  |  |
| Week 8 | 14 | -3.97 | 0.59 |  | 14 | | -0.70 | | 0.59 | |  |  | |  | |  | |  |  |
| Week 12 | 14 | -3.47 | 0.57 |  | 14 | | -1.13 | | 0.57 | |  | -2.35 | | [-4.00, -0.69] | | 0.007 | |  |  |
| IPSS-QOL |  |  |  |  |  | |  | |  | |  |  | |  | |  | |  |  |
| Week 4 | 13 | -1.19 | 0.38 |  | 14 | | -0.48 | | 0.38 | |  |  | |  | |  | |  |  |
| Week 8 | 14 | -1.50 | 0.36 |  | 14 | | -0.41 | | 0.36 | |  |  | |  | |  | |  |  |
| Week 12 | 14 | -1.14 | 0.34 |  | 14 | | -0.34 | | 0.34 | |  | -0.80 | | [-1.80, 0.20] | | 0.112 | |  |  |
| OAB-q total score |  |  |  |  |  | |  | |  | |  |  | |  | |  | |  |  |
| Week 4 | 14 | -21.69 | 4.41 |  | 14 | | -6.88 | | 4.32 | |  |  | |  | |  | |  |  |
| Week 8 | 14 | -29.06 | 4.34 |  | 14 | | -7.24 | | 4.34 | |  |  | |  | |  | |  |  |
| Week 12 | 14 | -31.56 | 4.56 |  | 14 | | -7.81 | | 4.56 | |  | -23.75 | | [-37.05, -10.44] | | 0.001 | |  |  |
| OAB-q symptom bother |  |  |  | |  |  | |  | |  | |  |  | |  | |  | | |
| Week 4 | 14 | -8.24 | 1.16 | |  | 14 | | -3.04 | | 1.13 | |  |  | |  | |  | | |
| Week 8 | 14 | -9.76 | 1.40 | |  | 14 | | -3.04 | | 1.40 | |  |  | |  | |  | | |
| Week 12 | 14 | -11.19 | 1.61 | |  | 14 | | -3.33 | | 1.61 | |  | -7.86 | | [-12.60, -3.11] | | 0.002 | | |
| OAB-q HRQoL |  |  |  | |  |  | |  | |  | |  |  | |  | |  | | |
| Week 4 | 14 | -13.42 | 3.73 | |  | 14 | | -3.93 | | 3.65 | |  |  | |  | |  | | |
| Week 8 | 14 | -19.21 | 3.28 | |  | 14 | | -4.28 | | 3.28 | |  |  | |  | |  | | |
| Week 12 | 14 | -20.29 | 3.29 | |  | 14 | | -4.57 | | 3.27 | |  | -15.71 | | [-25.31, -6.12] | | 0.003 | | |
| * *P*-values were derived from the cLDA model. Abbreviations: CI, confidence interval; IPSS, HRQoL, health-related quality of life; International Prostate Symptom Score, IPSS-QOL, quality of life index in IPSS, LSM, least square means; OAB-q, Overactive Bladder Questionnaire; OABSS, Overactive Bladder Symptom Score; SE, standard error | | | | | | | | | | | | | | | | | |  |  |
|  | | | | | | | | | | | | | | | | | |  |  |
|  | | | | | | | | | | | | | | | | | |  |  |
|  |  |  |  |  |  |  |  |  |  |  |  |  |  |  |  |  |  |  |  |
|  |  |  |  |  |  |  |  |  |  |  |  |  |  |  |  |  |  |  |  |
|  |  |  |  |  |  |  |  |  |  |  |  |  |  |  |  |  |  |  |  |
