# Supplementary material for: Efficacy and safety of Vibegron for the treatment of residual overactive bladder symptoms after laser vaporization of the prostate: A single‐center prospective randomized controlled trial (VAPOR TRIAL)
Source: Low Urin Tract Symptoms. 2024 Jul 2;16(4):e12529. doi: 10.1111/luts.12529 (PMC11500685; doi:10.1111/luts.12529)
Supplement: Supplementary file 3 — Table S3. Comparison of proportion of patients achieving MCIC for OABSS and MID for OAB‐q using multivariable logistic regression model. [file LUTS-16-e12529-s005.docx]

Table S3 Comparison of proportion of patients achieving MCIC for OABSS and MID for OAB-q using multivariable logistic regression model

| Variables | Reference | n | OR | 95% CI | *P*-value* |
| --- | --- | --- | --- | --- | --- |
| Achieving MCIC on OABSS (≥3-point decrease） | |  |  |  |  |
| Vibegron group | Follow-up group | 11/8 | 2.51 | [0.08, 1.95] | 0.256 |
| Achieving MID on OAB-q symptom bother (≥10-point decrease） | |  |  |  |  |
| Vibegron group | Follow-up group | 9/2 | 20.14 | [1.31, 308.91] | 0.031 |
| Achieving MID on OAB-q HRQoL (≥10-point decrease） | |  |  |  |  |
| Vibegron group | Follow-up group | 11/6 | 4.75 | [1.01, 21.35] | 0.042 |
| * *P*-values were derived from multivariable logistic regression model adjusted by age at consent and each baseline score. Abbreviation: CI, confidence interval; HRQoL, health related quality of life; OAB-q, overactive bladder questionnaire; OABSS, overactive bladder symptom score; OR, odds ratio | | | | | |
