# Supplementary material for: Efficacy and safety of Vibegron for the treatment of residual overactive bladder symptoms after laser vaporization of the prostate: A single‐center prospective randomized controlled trial (VAPOR TRIAL)
Source: Low Urin Tract Symptoms. 2024 Jul 2;16(4):e12529. doi: 10.1111/luts.12529 (PMC11500685; doi:10.1111/luts.12529)
Supplement: Supplementary file 4 — Table S4. Change from baseline for each endpoint (crude value). [file LUTS-16-e12529-s003.docx]

Table S4 Change from baseline for each endpoint (crude value)

|  | Group (FAS) | | | | | | |  |  | Group (PPS) | | | | | | |
| --- | --- | --- | --- | --- | --- | --- | --- | --- | --- | --- | --- | --- | --- | --- | --- | --- |
|  | Vibegron | | |  | Follow-up | | |  |  | Vibegron | | |  | Follow-up | | |
| Variables | *n* | Mean | SD |  | *n* | Mean | SD |  | Variables | *n* | Mean | SD |  | *n* | Mean | SD |
| Bladder diary |  |  |  |  |  |  |  |  | Bladder diary |  |  |  |  |  |  |  |
| 24-hour frequency | | | | | | | |  | 24-hour frequency | | | | | | | |
| Week 8 | 16 | -3.08 | 1.52 |  | 18 | 0.56 | 1.20 |  | Week 8 | 14 | -3.07 | 1.63 |  | 14 | 0.55 | 1.30 |
| Week 12 | 16 | -3.29 | 2.09 |  | 18 | 0.39 | 1.65 |  | Week 12 | 14 | -3.43 | 2.21 |  | 14 | 0.50 | 1.87 |
| 24-hour urgency | | | | | | | |  | 24-hour urgency | | | | | | | |
| Week 8 | 16 | -2.44 | 2.84 |  | 18 | -0.87 | 2.70 |  | Week 8 | 14 | -2.67 | 2.97 |  | 14 | -0.90 | 3.08 |
| Week 12 | 16 | -2.75 | 3.16 |  | 18 | -0.94 | 2.40 |  | Week 12 | 14 | -3.05 | 3.27 |  | 14 | -0.93 | 2.72 |
| 24-hour urgency incontinence episode | | | | | | | |  | 24-hour urgency incontinence episode | | | | | | | |
| Week 8 | 16 | -0.25 | 0.52 |  | 18 | 0.00 | 0.20 |  | Week 8 | 14 | -0.26 | 0.56 |  | 14 | -0.05 | 0.12 |
| Week 12 | 16 | -0.23 | 0.59 |  | 18 | 0.02 | 0.14 |  | Week 12 | 14 | -0.24 | 0.63 |  | 14 | 0.00 | 0.13 |
| Night-time frequency | | | | | | | |  | Night-time frequency | | | | | | | |
| Week 8 | 16 | -0.73 | 1.30 |  | 18 | 0.39 | 0.91 |  | Week 8 | 14 | -0.67 | 1.34 |  | 14 | 0.45 | 0.88 |
| Week 12 | 16 | -0.58 | 1.76 |  | 18 | 0.43 | 0.99 |  | Week 12 | 14 | -0.76 | 1.70 |  | 14 | 0.48 | 1.06 |
| Voided volume/micturition, mL | | | | | | | |  | Voided volume/micturition, mL | | | | | | | |
| Week 8 | 16 | 57.45 | 64.07 |  | 18 | -9.59 | 26.00 |  | Week 8 | 14 | 61.01 | 67.99 |  | 14 | -4.12 | 22.71 |
| Week 12 | 16 | 61.24 | 59.59 |  | 18 | -13.38 | 26.18 |  | Week 12 | 14 | 63.25 | 63.71 |  | 14 | -10.51 | 24.75 |
| OABSS total score | | | |  |  |  |  |  | OABSS total score | | | |  |  |  |  |
| Week 4 | 15 | -2.80 | 2.34 |  | 18 | -0.83 | 2.87 |  | Week 4 | 13 | -3.31 | 1.97 |  | 14 | -0.36 | 3.05 |
| Week 8 | 16 | -3.25 | 2.79 |  | 18 | -1.00 | 2.45 |  | Week 8 | 14 | -3.71 | 2.55 |  | 14 | -0.64 | 2.65 |
| Week 12 | 16 | -3.56 | 1.93 |  | 18 | -1.83 | 2.04 |  | Week 12 | 14 | -3.79 | 1.93 |  | 14 | -1.64 | 2.21 |
| IPSS total score | | | |  |  |  |  |  | IPSS total score | | | |  |  |  |  |
| Week 4 | 15 | -2.93 | 4.30 |  | 18 | -1.61 | 3.76 |  | Week 4 | 13 | -2.77 | 4.60 |  | 14 | -1.50 | 4.13 |
| Week 8 | 16 | -4.38 | 4.41 |  | 18 | -0.78 | 2.96 |  | Week 8 | 14 | -4.79 | 4.48 |  | 14 | -1.07 | 3.15 |
| Week 12 | 16 | -4.50 | 4.40 |  | 18 | -2.00 | 3.80 |  | Week 12 | 14 | -4.50 | 4.72 |  | 14 | -2.43 | 3.94 |
| IPSS voiding score | | | |  |  |  |  |  | IPSS voiding score | | | |  |  |  |  |
| Week 4 | 15 | -0.20 | 2.78 |  | 18 | -1.00 | 2.25 |  | Week 4 | 13 | 0.23 | 2.71 |  | 14 | -0.64 | 2.37 |
| Week 8 | 16 | -0.31 | 2.41 |  | 18 | -0.28 | 1.32 |  | Week 8 | 14 | -0.43 | 2.17 |  | 14 | -0.43 | 1.45 |
| Week 12 | 16 | -0.81 | 2.23 |  | 18 | -1.44 | 1.82 |  | Week 12 | 14 | -0.64 | 2.21 |  | 14 | -1.50 | 1.91 |
| IPSS storage score | | | |  |  |  |  |  | IPSS storage score | | | |  |  |  |  |
| Week 4 | 15 | -2.60 | 2.95 |  | 18 | -0.67 | 2.33 |  | Week 4 | 13 | -2.92 | 2.99 |  | 14 | -0.71 | 2.46 |
| Week 8 | 16 | -3.88 | 3.10 |  | 18 | -0.50 | 2.15 |  | Week 8 | 14 | -4.14 | 3.21 |  | 14 | -0.57 | 2.10 |
| Week 12 | 16 | -3.50 | 2.73 |  | 18 | -0.83 | 2.43 |  | Week 12 | 14 | -3.64 | 2.84 |  | 14 | -1.00 | 2.48 |
| IPSS-QOL | | | |  |  |  |  |  | IPSS-QOL | | | |  |  |  |  |
| Week 4 | 15 | -0.93 | 1.75 |  | 18 | -0.67 | 0.91 |  | Week 4 | 13 | -1.15 | 1.77 |  | 14 | -0.50 | 0.94 |
| Week 8 | 16 | -1.38 | 1.67 |  | 18 | -0.56 | 0.78 |  | Week 8 | 14 | -1.50 | 1.74 |  | 14 | -0.43 | 0.85 |
| Week 12 | 16 | -1.06 | 1.53 |  | 18 | -0.33 | 0.84 |  | Week 12 | 14 | -1.14 | 1.61 |  | 14 | -0.36 | 0.93 |
| OAB-q total score | | | |  |  |  |  |  | OAB-q total score | | | |  |  |  |  |
| Week 4 | 15 | -21.00 | 23.11 |  | 18 | -4.94 | 11.79 |  | Week 4 | 13 | -22.15 | 24.55 |  | 14 | -6.29 | 12.45 |
| Week 8 | 16 | -27.50 | 26.52 |  | 18 | -6.67 | 9.70 |  | Week 8 | 14 | -29.79 | 27.01 |  | 14 | -6.64 | 10.54 |
| Week 12 | 16 | -30.63 | 26.12 |  | 18 | -6.44 | 15.40 |  | Week 12 | 14 | -32.29 | 27.08 |  | 14 | -7.21 | 15.45 |
| OAB-q symptom bother | | | |  |  |  |  |  | OAB-q symptom bother | | | |  |  |  |  |
| Week 4 | 15 | -8.93 | 6.77 |  | 18 | -1.39 | 4.13 |  | Week 4 | 13 | -9.46 | 7.08 |  | 14 | -2.00 | 4.40 |
| Week 8 | 16 | -10.31 | 8.99 |  | 18 | -1.94 | 5.09 |  | Week 8 | 14 | -10.86 | 9.36 |  | 14 | -2.00 | 5.52 |
| Week 12 | 16 | -11.63 | 9.45 |  | 18 | -1.56 | 7.00 |  | Week 12 | 14 | -12.29 | 9.84 |  | 14 | -2.29 | 7.71 |
| OAB-q HRQoL | | | |  |  |  |  |  | OAB-q HRQoL | | | |  |  |  |  |
| Week 4 | 15 | -12.07 | 18.32 |  | 18 | -3.56 | 10.29 |  | Week 4 | 13 | -12.69 | 19.6 |  | 14 | -4.29 | 10.99 |
| Week 8 | 16 | -17.19 | 18.87 |  | 18 | -4.72 | 7.70 |  | Week 8 | 14 | -18.93 | 19.14 |  | 14 | -4.64 | 7.20 |
| Week 12 | 16 | -19.00 | 17.85 |  | 18 | -4.89 | 10.91 |  | Week 12 | 14 | -20.00 | 18.54 |  | 14 | -4.93 | 9.76 |
| Abbreviations: FAS, Full Analysis Set; HRQoL, health-related quality of life; IPSS, International Prostate Symptom Score; IPSS-QOL, quality of life index in IPSS; LSM, least square means; OAB-q, Overactive Bladder Questionnaire; OABSS, Overactive Bladder Symptom Score; PPS, Per Protocol Set; SD, standard deviation | | | | | | | | | | | | | | | | |
