# Supplementary material for: Efficacy and safety of Vibegron for the treatment of residual overactive bladder symptoms after laser vaporization of the prostate: A single‐center prospective randomized controlled trial (VAPOR TRIAL)
Source: Low Urin Tract Symptoms. 2024 Jul 2;16(4):e12529. doi: 10.1111/luts.12529 (PMC11500685; doi:10.1111/luts.12529)
Supplement: Supplementary file 5 — Table S5. Change in mean 24‐h frequency from baseline to 12 weeks in a population subgrouped by patients' background. [file LUTS-16-e12529-s001.docx]

Table S5 Change in mean 24-hour frequency from baseline to 12 weeks in a population subgrouped by patients’ background

|  |  |  | Group | | | | | | |
| --- | --- | --- | --- | --- | --- | --- | --- | --- | --- |
|  |  |  | Vibegron | | |  | Follow-up | | |
|  |  |  | N | Mean | SD |  | N | Mean | SD |
| Change in mean 24-hour frequency at 12 w, times | | |  |  |  |  |  |  |  |
|  | Age, years | < 65 | 1 | -3.33 | - |  | 2 | -1.00 | 0.47 |
|  |  | ≥ 65 | 15 | -3.29 | 2.16 |  | 16 | 0.56 | 1.66 |
|  |  | < 75 | 6 | -4.50 | 2.16 |  | 8 | 0.21 | 1.10 |
|  |  | ≥75 | 10 | -2.57 | 1.76 |  | 10 | 0.53 | 2.03 |
|  | Vaporization | PVP | 7 | -2.52 | 1.82 |  | 8 | -0.25 | 1.27 |
|  |  | CVP | 9 | -3.89 | 2.18 |  | 10 | 0.90 | 1.79 |
|  | 24-hour frequency | < 11 | 4 | -1.92 | 2.01 |  | 8 | 0.67 | 2.01 |
|  |  | ≥ 11 | 12 | -3.75 | 1.98 |  | 10 | 0.17 | 1.36 |
|  | OABSS total score | < 6 | 2 | -3.50 | 0.24 |  | 4 | 0.42 | 1.32 |
|  |  | ≥ 6 | 14 | -3.26 | 2.24 |  | 14 | 0.38 | 1.77 |
|  | IPSS storage score | < 8 | 2 | -1.50 | 0.71 |  | 4 | -0.08 | 2.10 |
|  |  | ≥ 8 | 14 | -3.55 | 2.10 |  | 14 | 0.52 | 1.56 |
| Abbreviations: CVP, contact laser vaporization of the prostate; IPSS, International Prostate Symptom Score; OABSS, Overactive Bladder Symptom Score; PVP, photo-selective vaporization of the prostate; SD, standard deviation; w, week | | | | | | | | | |
